# Exploring the role of structuralist methodology in the neuroscience of consciousness: a defense and analysis

**DOI:** 10.1093/nc/niad011

**Published:** 2023-05-17

**Authors:** Lukas Kob

**Affiliations:** Philosophy Department, Otto-von-Guericke-University of Magdeburg, Magdeburg, Germany; Humboldt-Universität zu Berlin, Research Training Group 2386 “Extrospection”, Berlin, Germany; Bernstein Center for Computational Neuroscience, Charité—Universitätsmedizin, Berlin, Germany; Faculty of Philosophy, Berlin School of Mind and Brain, Humboldt-Universität zu Berlin, Berlin, Germany

**Keywords:** structuralism, quality space, quality space theory, consciousness, representational geometry, relational geometry, NCC

## Abstract

Traditional contrastive analysis has been the foundation of consciousness science, but its limitations due to the lack of a reliable method for measuring states of consciousness have prompted the exploration of alternative approaches. Structuralist theories have gained attention as an alternative that focuses on the structural properties of phenomenal experience and seeks to identify their neural encoding via structural similarities between quality spaces and neural state spaces. However, the intertwining of philosophical assumptions about structuralism and structuralist methodology may pose a challenge to those who are skeptical of the former. In this paper, I offer an analysis and defense of structuralism as a methodological approach in consciousness science, which is partly independent of structuralist assumptions on the nature of consciousness. By doing so, I aim to make structuralist methodology more accessible to a broader scientific and philosophical audience. I situate methodological structuralism in the context of questions concerning mental representation, psychophysical measurement, holism, and functional relevance of neural processes. At last, I analyze the relationship between the structural approach and the distinction between conscious and unconscious states.

## Introduction

In the quest to unravel the neural correlates of consciousness (NCC) (Crick and Koch 1990, Chalmers 2000, Koch 2004), traditional methodology in consciousness research has faced significant challenges. This methodology typically contrasts conscious and possibly unconscious perceptual states and draws inferences about the neural basis of this contrast (Lepauvre and Melloni 2021). However, this approach has not led to convergence of measures, despite its widespread use over the years. Different research groups have focused on different operationalizations of the conscious/unconscious contrast, leading to divergent theories and findings (Irvine 2012).

Structuralism provides an alternative approach to unravel the neural basis of phenomenal consciousness. Rather than contrasting conscious and unconscious states, this approach describes conscious states in terms of their relational structure (Haynes 2009, Opie and O'Brien 2015, Fink et al. 2021, Malach 2021, Tsuchiya and Saigo 2021, Lau et al. 2022, Lyre 2022). One way to describe the structure of experience is through the use of quality spaces, which depict the relationships of similarities and differences among basic sensory qualities (Rosenthal 2010, 2015). In a color quality space, for instance, perceivable colors are arranged along dimensions of hue, saturation, and lightness (Kuehni 2003). Structuralists then aim to unravel the neural encoding of characteristic structural properties of phenomenal experiences, such as color structure. By examining the neural correlates of these structural properties, structuralism offers a promising avenue for understanding the mechanisms underlying conscious experience.

To study the neural encoding of the structure of quality spaces, researchers focus on neural structures that exhibit structural similarities to these quality spaces (Haynes 2009, Opie and O’Brien 2015, Fink et al. 2021, Malach 2021, Tsuchiya and Saigo 2021, Lau et al. 2022, Lyre 2022). One commonly used approach involves examining neural pattern-similarity structures in the state spaces of neural systems (Churchland and Sejnowski 1992) that mirror qualitative structures. The structure of neural processing has been assumed to be intricately tied to conscious experience. As Opie and O’Brien (2015: 459) argue, “it is in virtue of possessing certain structural properties that a pattern of neural activity constitutes a conscious experience.” Relatedly, Malach (2021: 6, Fig. 3) writes that “conscious content is defined by neuronal pattern-similarity distances.”

The methodological approach of mapping representational spaces (Kriegeskorte and Kievit 2013) is often intertwined with broader philosophical problems regarding structuralism. Structuralism as a broader philosophical position typically assumes that relations are important in defining entities and has been extensively debated in both philosophy of mind (Isaac 2014, Opie and O'Brien 2015, Morrison 2020) and philosophy of science (Ladyman 1998). In these debates, structuralism has faced criticism related to questions of representation (Suárez 2003) and holistic definitions (Fodor and Lepore 1992). In this paper, I aim to defend structuralism as a methodological approach. In my view, some philosophical issues related to structuralism do not necessarily threaten the use of structuralism as a methodological approach. However, in the context of methodology, challenges arise concerning psychophysical measurement and the relevance of state space structures to neural processing which I will address. Additionally, the explicit relationship between structuralist methodology and traditional methodology in consciousness science is yet to be thoroughly explored. To address this concern, I relate structuralism to more traditional approaches to consciousness science.

The discussion proceeds as follows: in the section “The contrastive approach in consciousness science”, I provide a brief overview of the empirical challenges associated with measuring consciousness and how structuralism might offer an alternative approach to address these challenges and advance our understanding of consciousness. The section “The methodology of structuralism” introduces the structuralist methodology used in psychophysics and systems neuroscience to map quality spaces to neural state spaces. In the section “A defense of the structural approach”, I delve into the topics of representation, psychophysics, and holism and provide an account of the functional relevance of neural pattern-similarity structures. Finally, I examine the relationship between the structuralist methodology and the conscious/unconscious distinction in the section “The specificity of neural pattern-similarity strucures related to consciousness”. By providing a comprehensive overview of structuralist methodology and its potential application to consciousness studies, this paper aims to contribute to the emerging structuralist research program in consciousness studies.

## The contrastive approach in consciousness science

The search for the neural basis of consciousness has been a central focus in the field of neuroscience since the early 1990s under the heading of the quest for finding the “NCC” (Chalmers 2000, Fink 2016). Pioneered by Crick and Koch (1990) (also see Koch 2004), this research program aims to pin down the neural areas that correlate with the occurrence of consciousness. The goal is to gather evidence on the complex phenomenon of consciousness, independent of any metaphysical considerations. However, the notion of neural “correlate” is not fully appropriate, as many scholars in the field rather look for neural processes that explain consciousness (Wu 2018) and hence are not only correlated with consciousness but minimally sufficient for its occurrence under the right background conditions (Chalmers 2000). Delineating the NCC from the background conditions has also been understood as searching the “core” NCC.

Methodologically, the study of consciousness in neuroscience relies on the distinction between general perceptual functioning and conscious phenomenal experience. The aim is to identify the neural areas that specifically relate to conscious experience as opposed to mere unconscious perception. The field uses controlled experiments to investigate the relationship between perception and experience (Kim and Blake 2005). Researchers manipulate conscious experience independent of perception using various psychophysical methods such as masking, which involves presenting a stimulus above the perceptual threshold but suppressing it so that it is not consciously experienced, although the subject can still respond to it. This indicates that the stimulus is still perceived, but not consciously experienced. Other methods used include priming, continuous flash suppression, change blindness, and inattentional blindness (Kim and Blake 2005).

Further experimental approaches to distinguishing conscious experience from sensory input use ambiguous imagery or binocular rivalry (Kim and Blake 2005). These techniques take advantage of the fact that humans can only perceive one coherent interpretation of an ambiguous image or one stimulus presented to each eye at a time. In ambiguous imagery, a stimulus contains two possible coherent interpretations, and our experience switches back and forth between them. In binocular rivalry, a stimulus is presented to each eye separately, causing the subjective experience to switch between the input from one eye or the other. For example, decoding stable percepts in binocular rivalry can provide evidence on NCCs (Haynes and Rees 2005). Clinical and lesion studies also offer crucial evidence for the dissociation of perception and consciousness. Certain neurological conditions, such as blindsight cases (Weiskrantz 1998), where patients have damage to part of the visual cortex but still respond to stimuli despite claiming not to see it, and certain forms of epilepsy (Searle 1992), provide further examples of behavior occurring without conscious phenomenal experience.

Despite the extensive use of various experimental methods to study consciousness, the field of consciousness science faces controversies and challenges. One ongoing debate concerns the validity of blindsight patients’ reports of conscious experience (Alexander and Cowey 2010, Danckert et al. 2021, Derrien et al. 2022), with some papers calling into question their inability to phenomenally experience visual stimuli (Phillips 2021a, 2021b; see Michel 2022 for reply). Furthermore, questions remain about whether the methods used to manipulate conscious experience genuinely affect conscious experience or merely the neural processes involved in reporting or introspecting on it (Block 2019, Michel and Morales 2020). Even methods such as binocular rivalry, which are commonly used to manipulate conscious experience, have been called into question, as there is evidence of “unconscious” binocular rivalry, suggesting that the mechanisms underlying the switch of perceptions may not be sufficient for conscious experience (Zou et al. 2016; also see Lin and He 2009). Additionally, the different methods of manipulation used in the field may target different underlying factors, making it challenging to draw inferences between studies that use different techniques (Tulver 2019). Therefore, it remains a significant challenge to accurately “distill” (Aru et al. 2012) consciousness as a unified scientific phenomenon from other perceptual and cognitive processes (Irvine 2012, Lepauvre and Melloni 2021).

Overcoming the challenges associated with measuring consciousness may be facilitated by focusing on the structure of experience. This is because structuralism does not contrast conscious and unconscious perception, but rather aims to describe the structural properties of phenomenal experience. By looking for neural systems that give rise to these structural properties, NCCs can be identified. The structural approach uses templates based on quality space descriptions, providing an additional method to search for neural systems encoding phenomenal experience. As a result, the structural approach has the potential to provide a complementary approach to traditional experimental methods in the study of consciousness and may open up new avenues for future research. In the next section, I will present this methodology in detail.

## The methodology of structuralism

### Quality spaces

The structures of sensory experience are scientifically described as quality spaces (Clark 1993, 2000, Rosenthal 2010, 2015), with color spaces being the most extensively studied example. Color spaces depict color relationships in a geometric manner. In a schematic view of the color cone (Churchland 2005), a polar axis represents the hue dimension. Purple is perceived as similar to both blue and red and thereby connects the ends of the visible spectrum, around 400 and 700 nm, respectively, in a way that transforms the linear shape of the physical light wave spectra into a polar dimension (Churchland 2007, see also Ramanath et al. 2004). The experienced brightness of colors is typically represented as the vertical axis and saturation as the horizontal axis. Figuratively speaking, as you move up the color space, the colors get brighter until you reach the white point. The further you move away from the center, the gray point, the more saturated the colors become. As you move through the polar dimension, you see the hues ordered by their similarity, e.g. after red comes orange, then yellow, and so on. The hues that are opposite to each other on the color wheel are maximally dissimilar.

Quality spaces offer a unique perspective into the study of phenomenal consciousness. Psychophysicists have created various color spaces that showcase the relationships between colors in a geometric fashion (for an overview, see Kuehni 2003, Kuehni and Schwarz 2008). While there are some differences between these color spaces, the solids modeled in color spaces share similar structural properties such as being three-dimensional and possessing an asymmetric shape. (For an overview on kinds of color spaces, see Thompson et al. 1992: 360; Kuehni 2003: 357.) This particular shape is due to the fact that human color perception is limited and does not occur for all possible coordinates of space. For instance, yellow hues can be lighter than blue hues and vice versa, leading to a distorted and asymmetrical shape of the color space. These peculiarities of human color perception are represented in the specific structural properties of a specific domain of phenomenal consciousness.

Rosenthal’s (2010, 2015) quality space theory is a comprehensive framework that aims to encompass all sensory modalities. Geometrical models have been developed to describe quality spaces for various sensory experiences, such as pitch (Shepard 1982, Janata 2008, Collins et al. 2014), gustatory experiences (Churchland 1986: 302; Gärdenfors 2000), and olfaction (Howard et al. 2009, Koulakov et al. 2011, Young et al. 2014, but see also Jraissati and Deroy 2021). While Rosenthal’s theory focuses on “basic” sensory experiences, it remains unclear whether quality spaces should be limited to such experiences or whether the notion of “qualitative dimension” can extend to any geometrical ordering of subjective experiences related to stimuli, such as faces (Grossman et al. 2019), or object domains, such as cars (Bellmund et al. 2018). To account for the diverse range of stimuli domains used to evaluate the structure of subjective experiences in scientific practice, I use the term “quality space” in a broad sense to refer to all kinds of orderings of subjective appearances, whether basic or composite.

### Neural spaces

The study of neural processes underlying quality spaces, such as color space, involves identifying neural pattern-similarity structures that correspond to the structural properties of the quality space. This can be accomplished by characterizing the structure of neural processing in terms of the similarity relations among neural firing patterns. These relations can then be used to construct a neural similarity space, where each activation unit (assumed to be an individual neuron) represents a dimension, and a complete system state is represented as a point in the space (Churchland and Sejnowski 1992). The distances between these points indicate the similarity of corresponding system states. Notably, activation patterns of neural spaces are limited to a specific area within the space, referred to as the neural manifold, due to the specific wiring of the system (Jazayeri and Afraz 2017). This limitation is a crucial aspect of the activation space framework, as neural networks adapt their distribution and strength of synaptic connections to meet the demands of the system. Networks trained on specific stimulus structures often exhibit similar neural manifold structures, even if the underlying distribution of connection strengths is different (Grossman et al. 2019).

While formulating a structural similarity relationship would require a mathematical mapping between quality spaces and neural spaces, formulating such a precise mapping (a mathematical morphism or even functor; Tsuchiya and Saigo 2021) is often not scientifically feasible (Kriegeskorte and Kievit 2013: 401–402). One approach to study the structural similarity between quality spaces and neural spaces is to compare dimensionality reduced subsets of quality and neural spaces. In order to do so, a high-dimensional neural space measured using neuroimaging methods such as functional magnetic resonance imaging (fMRI) has to be reduced in its dimensions. The goal of dimensionality reduction is to find the most important dimensions in the voxel space that correspond to the dimensions in the psychological color space. The statistical methods used in neuroscience involve reducing the number of dimensions in a data space to explain its variability. One common method used is principal component analysis (PCA), which orders the eigenvectors of the covariance matrix based on their eigenvalues (Jolliffe and Cadima 2016). The eigenvectors with the highest eigenvalues, which represent the directional vectors of the data cloud, explain a large part of the variability. PCA allows for the transformation of the distances in a high-dimensional space into a low-dimensional space using the eigenvectors as the basis vectors. Dimensionality reduction of neural spaces allows for a comparison with low-dimensional quality spaces.

A study that illustrates this methodology well is the study by Brouwer and Heeger (2009) (see also Brouwer and Heeger 2013). They use a substructure from the Commission Internationale de l'Eclairage (CIE) L*a*b* space (CIE 1986) and relate it to neural pattern-similarity spaces in the visual cortex. It was found that eight equally distant, equally saturated, and bright color stimuli form a color-circle-like pattern in the principal component space of V4. This type of “encoding” (Haynes 2009) implies that the neural patterns evoked by a red experience are similar to those evoked by an orange experience but different from those associated with green experiences, consistent with the structure of our subjective experiences of color. Dimensionality-reducing methods are often criticized due to the inherent degree of freedom in selecting the number of dimensions. As a result, it is not guaranteed that the neural system actually operates based on these dimensions and that the same number of dimensions implies the processing of the same features. Therefore, caution should be exercised when interpreting the results obtained from using these methods (Goddard et al. 2018).

Representational similarity analysis (RSA) is a widely used method that circumvents assumptions about the dimensionality of quality spaces and neural spaces (Laakso and Cottrell 2000, Kriegeskorte et al. 2008, Kriegeskorte and Mur 2012, and Kriegeskorte and Kievit 2013 provided a predecessor). RSA utilizes representational dissimilarity matrices (RDMs) to calculate the dissimilarity between neural patterns or psychological states in response to different stimulus conditions. The stimuli are represented as both rows and columns in the RDM, whereas the cells contain the degree of dissimilarity between each pair of stimuli, measured based on the applied similarity metric and measurement method (such as psychological ratings or activation pattern similarity). The structural similarity between different RDMs can be measured by correlating the matrices, providing an elegant means to investigate the structural similarity assumption without the need to make assumptions about underlying dimensions or provide a mapping function. Studies using RSA have provided support for the mapping of quality spaces and neural spaces (Bohon et al. 2016, Cichy et al. 2019, Grossman et al. 2019, Kim et al. 2020, Rosenthal et al. 2021).

### Structural constraints on NCCs

The search for neural systems that encode the structure of qualitative consciousness can contribute to single out the neural correlates of phenomenal consciousness and its function from a different angle. This approach has been adopted by various neuroscientists. Tsuchiya et al. (2020: 5) note that there “is clearly a need to extract the structure from neural activity to allow for testing of hypothetical isomorphisms between neural and phenomenal structures.” In line with this, Haynes (2009: 198) claims that

a systematic mapping between states of the core NCC and conscious experiences […] would have additional explanatory power. It could explain how similarities or relationships between different sensations are encoded by similar relationships between states of the core NCC, as in the case of perceptual spaces.

In a previous paper (Fink et al. 2021), my colleagues and I argued that the mapping between structures of phenomenal consciousness and neural pattern-similarity structures can be exploited for distinguishing conscious and unconscious neural system states because there are systems that process stimulus information but show no structural similarity to the special structure of consciousness. For example, the processing of color information in the visual cortex shows differences in activation space structures between V1 and V4 (Brouwer and Heeger 2009, 2013). While colors can be decoded linearly from V1 (see also Parkes et al. 2009), the hue arrangement in the activation space structure is different from the way we experience colors. However, in V4, colors can be decoded and the activation space structure aligns with the color wheel, suggesting a closer match between neural activation and phenomenal experience (on the relationship between decoding and activation space structures, see, e.g. Kriegeskorte and Diedrichsen 2019). Therefore, from a structuralist perspective, V4 is an NCC candidate, whereas V1 is not, even though V1 does process color information.

A mismatch between the topological structure of information in neural pattern-similarity space and the structure of conscious experience supports the exclusion of neural areas that process stimulus information but lack a corresponding activation space structure from consideration as potential correlates of consciousness. By applying the principle of aligning activation space structures with conscious experience, we formulated a ‘structural similarity constraint’ for identifying such neural correlates:

Neural substrates of phenomenal types must share the structure governing the phenomenal types they are associated with, i.e., the structure of a phenomenal space. (Fink et al. 2021: 15)

The structural approach offers a promising strategy for addressing methodological challenges in consciousness research. One key advantage is that it does not rely on subjective reports of consciousness awareness, which can be difficult to validate: we do not have to worry whether subjects “really” had a phenomenal experience of a stimulus. Instead, by assessing the structure of experience under experimental conditions in which the stimuli are presented above the perceptual threshold, we can measure the structure of phenomenal consciousness without relying on contrasting conscious awareness from unconscious perception.

## A defense of the structural approach

Although many have embraced a structuralist approach, its application in the field of consciousness science is not without its challenges. State space accounts have been criticized in terms of their implications regarding mental representation and meaning holism, and the measurement of quality spaces is entangled with fundamental debates in psychophysics. Additionally, structural similarity is a fairly unconstrained relation and the relationship between neural activation structures and neural processing is not well defined, which raises questions about the relevance of structural approaches. By addressing these challenges and presenting original perspectives, this paper aims to contribute to the establishment of structuralism as a valuable framework in the study of consciousness.

### Representation

In the context of RSA and the study of “representational geometries” (Kriegeskorte and Kievit 2013), the term “representation” is central, as the methods aim to map “representational spaces.” As a result, RSA is often interpreted within the framework of mental representations (Roskies 2021). However, this interpretation is not straightforward. While “representational geometry probably reflects the statistical structure of the world” (Kriegeskorte and Wei 2021: 715), this alone does not necessarily support a robust representational relationship between the neural activation structure and structures in the external world.

Demands on a robust notion of representation involve a clear understanding of how a representation is related to external objects and events through the specific logic of a representational relation (Goodman 1976), how a representation can function as an internal surrogate, and how misrepresentations can occur. Ramsey (2007) identifies such constraints as the “job description challenge” for any theory of representation. A representational account of neural pattern-similarity structures requires showing how these meet these demands on a theory of representation. Although a focus on neural structures suggests a structural notion of representation (representation by structural similarity to the environment) (Shagrir 2012), state space structures have also been interpreted as indicator representations (representation by correlation with environmental events) (Ramsey 2007). Additionally, deflationists who hold a view of representation in cognitive science deny the idea that cognitive science involves a strong notion of representation. They instead interpret a more pragmatic notion that only includes the mathematical content of cognitive models and the scientist’s interpretation in the context of experiments (Egan 2020). A critic of the structural approach may argue that structuralists lack a coherent theory of representation, rendering the notion of “representational structure” ambiguous and the research program of structuralism unclear.

However, when investigating the structural similarity between the neural encoding of consciousness and the structure of phenomenal experience, the representational properties of neural and phenomenal structures are not directly relevant. This is because the mapping of qualitative and neural structures can be studied in terms of two different research questions: first, exploring the relationship between psychological and neural structures in the tradition of Fechner’s (1860) inner psychophysics and second, investigating the extent of structural similarity between neural structures and external structures, which would constitute a representationalist research program. When exploring the relationship between consciousness and the brain, we take part in the former approach. Therefore, a structuralist methodology in consciousness science can be developed without an account of mental representation and the lack of such an account is not a flaw for the methodological approach in the context of consciousness science. To sidestep complex questions about state space representation, I rely on the more neutral term “neural pattern-similarity structure” rather than “representational geometry” or “representational space.”

### Structural similarity is “cheap to have”

While structural approaches hold great potential for advancing our understanding of consciousness, they face a significant challenge outlined by Newman’s critique of Russell’s structuralism (Newman 1928). Newman argued that any two sets of elements can be arranged to exhibit structural similarity, rendering claims about neurophenomenal structural similarity potentially trivial or imprecise. The complexity of the brain’s structural properties only amplifies this issue, given the seemingly endless ways to arrange neurons and identify structural properties.

To address this challenge, Russell proposed distinguishing between relevant and accidental structural similarities. Analogously, in the context of mapping qualitative spaces onto neural spaces, we understand the relevant structural relations as qualitative similarity relations and neural pattern-similarity distances in state space. Therefore, mapping is not arbitrary, but rather a specific structural similarity is being assessed. However, selecting appropriate neural similarity metrics is no simple task, as the vast array of possible metrics and the constraints they impose must be carefully balanced (see Walther et al. 2016, Charest et al. 2018, Bobadilla-Suarez et al. 2020 for more on this topic). Nonetheless, the structuralist methodology avoids the criticism that “structural similarity is cheap to have” by focusing on mapping specific structures, namely, qualitative structures and neural pattern-similarity structures. But measuring qualitative structures is no easy task either.

### Measuring qualitative structures

Critics of the structural approach may also argue that there is no direct way to measure the subjective properties of qualitative experience. Psychophysics has long been plagued by controversy regarding the best measure to capture the metrics of subjective experience (Wagenaar 1975, see also Gescheider 1997, Lawless 2013). Discrimination abilities, such as just noticeable differences, have been criticized as potentially reflecting perceptual rather than consciousness-related metrics, as perceptual distinctions can be more fine-grained than differences in subjective experience. To address this issue, similarity measures were advocated as a more direct measurement of experience structures (Stevens 1957).

However, a classical criticism of similarity judgments is that they do not meet the mathematical requirements for being a metric in a geometrical sense, due to their asymmetric and context-dependent nature (Tversky 1977). To overcome Tversky’s criticism, flexible models of similarity structures that allow for “flexible selection and weighting of the dimensions of the space” have been proposed (Kriegeskorte and Mur 2012: 1) to account for context dependency. Feature weighting, in particular, plays a central role in newer iterations of RSA methodology (Kaniuth and Hebart 2022). Another concern with using subjective similarity ratings is the potential for subjects to misintrospect their own phenomenal metrics and therefore fail to appropriately rate their experienced similarity. To address this issue, scientists employ more “intuitive” assessments of subjective similarity that plausibly do not hinge on as many postperceptual processes as explicit similarity judgments, such as measuring reaction times (Cohen et al. 2015, 2017) or arranging stimuli in space to serve as proxies for experienced similarities (Kriegeskorte and Mur 2012).

To capture measures of phenomenal similarity, traditional psychophysics and tasks related to conscious awareness can be usefully combined. For example, two studies by Cohen et al. (2015) utilized an odd-one-out task combined with experimental paradigms related to conscious awareness, such as masking and continuous flash suppression. In this task, participants were presented with a target stimulus from one category, which was masked by a series of stimuli from a different category, and had to identify the target stimulus. Response time was used as a measure of experienced similarity, measured by how long it took on average for participants to detect a target stimulus of one category among masks of a different category, with correct identifications in 80% of the cases. This yielded a characteristic distribution of response times among the category pairs. For example, cars were harder to detect among houses than faces among houses. This task provides a measure of how long it takes to discriminate the appearance of stimuli in consciousness and is therefore an interesting example of how relational properties of subjective experience can be measured using reaction times combined with more awareness-related tasks.

Although direct measurement of phenomenal consciousness is not (yet) possible, psychophysical methods that aim at the qualitative structure of experience can provide an approximate measure of the structure of conscious experience. These offer an addition to approaches that focus only on differences between conscious and unconscious processing. This allows valuable insights into the structural properties of consciousness and its underlying neural substrates. However, even after formulating and measuring the specific structures, the identification of elements within these structures can still pose further problems. This has been discussed under the label of holism.

### Holism

In neurophilosophy, structural approaches are typically associated with holism, which posits that the meanings of neural patterns are determined by their location in state space (Churchland 1993, 1998). However, this approach has faced criticism for suggesting that individuals with different semantic spaces would differ in all their meanings, making all holistically defined meanings idiosyncratic (Fodor and Lepore 1992). As meanings are publicly shared, holism fails at offering an account on neurosemantics. This issue is also relevant to the mapping of quality spaces to neural spaces, where the fit of two similarity structures is generally associated with a holistic perspective on sensory qualities and neural patterns (Clark 2000, Fink et al. 2021, Lyre 2022). A holistic or structural definition identifies an object based on its relational properties, such as its position within a relational structure. For example, defining a color quality as a coordinate in color space amounts to a structural description that is holistic (Clark 2000, Rosenthal 2010, 2015, Fink et al. 2021, Lyre 2022).

I have four points to make in response to this. First, the neurophenomenal structural similarity hypothesis and the search for neural encodings of quality spaces do not require a strong holistic theory of phenomenal qualities and neural coding. Instead, a methodological form of holism can be applied, which assumes that the elements of both domains, regardless of their nature or semantics, can be sufficiently ‘individuated’ based on their relationships. This leaves open whether and how neural activation patterns can convey meaning and how phenomenal character is determined, but still allows the search for structural similarities. While I have a favorable view of phenomenal structuralism, holism poses an example where the methodological aspect of searching for structural similarities between neural and phenomenal domain rests on less strong premises than structuralism as a stance on phenomenal consciousness more broadly. Interested readers can explore related work on structuralism, such as the works of Clark (2000), Rosenthal (2010), Chalmers (2012), Isaac (2014), Rosenthal (2015), Morrison (2020), Fink et al. (2021), and Lyre (2022).

Second, it is important to note that structural similarity is not an all-or-nothing affair. This is already reflected in RSA (Kriegeskorte et al. 2008), which provides a measure of the degree of the overall structural similarity between two domains (see also Laakso and Cottrell 2000). Interindividual differences in state space structures do not necessarily imply a complete mismatch between the corresponding neural spaces. Recent investigations have made progress in comparing state space distances between individuals, such as through hyperalignment, which involves embedding neural spaces in a higher-dimensional space (Haxby et al. 2020; see also Kawakita et al. 2023).

Third, interindividual comparisons have revealed a more nuanced picture than some critics have suggested. Some results suggest that there are shared structural properties of representational spaces, even though there is also interindividual variability due to fine-tuning to individual experiences and plausibly genetic factors (Charest et al. 2014). In this sense, neural spaces can be viewed as possessing commonly shared structural properties, with some variation due to individual differences.

Fourth, it is important to note that mapping subjective qualities does not face the same constraints as defining meaning. In fact, finding interindividual differences in subjective experience of qualities is a desired outcome and might be stronger than semantics (Fink et al. 2021), as individual differences in perception are well-established (Hardin 2014). It may well be that there are major differences in color experience that are nevertheless not noticeable in everyday life, where the rough categories that color terms have are sufficient for pragmatic purposes. All in all, holistically mapping quality spaces to neural spaces is a suitable way to investigate the neural encoding of subjective qualities.

Qualitative states and their corresponding neuronal activation patterns can be related based on their respective structural properties, such as their coordinates in qualitative and neuronal space. This approach does not assume that the location in the structure defines the qualitative states or that neuronal patterns derive their meaning solely from their location in the activation space. It is possible that the mapping of individual states may fail if the structural similarity is not fully satisfied. However, the structural approach offers a way to filter out neuronal systems that demonstrate a strong degree of structural similarity to qualitative structures. This is already a methodological achievement. Nevertheless, one could argue that this mapping may not be relevant to neural processing at all and may be purely accidental rather than functional in neural processing.

### Neural pattern-similarity structures describe functional similarities

Although structural similarities between quality spaces and neural pattern-similarity spaces have been observed, it can be questioned whether such activation geometries are functional in neural processing or whether the correlations found are merely accidental properties. Essentially, we do not yet know whether the structural similarity between quality spaces and neural pattern-similarity structures has any relevance.

However, considering qualitative structures as encoded in the functional structure of neural processing suggests that the structure of subjective qualities is not accidental, but matters in neural processing. In addition to the ample evidence on the structural similarities between qualitative and neural states, such as the reviews by Kriegeskorte and Kievit (2013) and recent studies by Cichy et al. (2019) and Grossman et al. (2019) show, there are three additional sources of evidence supporting this claim.

First, studies by Cohen et al. (2015, 2017) show that activation space structures are statistically linked to behavioral patterns. In an odd-one-out task, response times were used to measure the experienced similarity of different categories (see earlier), and the structure of neural pattern-similarity structures was found to explain this structure of reaction times related to distinguishing category pairs. As brain signals precede behavior, it is a first hint that the structure of neural pattern-similarities in visual cortex could be causally involved in producing corresponding behavioral patterns.

Second, interindividual differences provide another route of evidence for the functional properties of neural pattern-similarity structures. Comparing the fit between neural pattern-similarity structures and perceptual behavior between individuals reveals that an individual’s own neural pattern-similarity structure explains individual components of behavioral structures, while commonly shared behavioral patterns are explained by commonly shared neural pattern-similarity structures (Charest et al. 2014). This suggests that varying the structural properties of neural pattern-similarity structures goes hand in hand with corresponding changes in behavioral patterns, suggesting a functional role of the former in determining the latter.

Third, convergent evolution provides additional support for the functional relevance of neural pattern-similarities. Grossman et al. (2019) compared the activation geometries in the middle layers of deep convolutional neural networks (DCNN) with human neural pattern-similarity structures related to the same face stimuli and found striking second-order similarities that emerged from training on the same stimuli. Malach (2021: 8) interprets these findings within the framework of convergent evolution in biology.

Imagine an alien that knows nothing about flying or aerodynamics, examining a range of flying creatures of different kinds—both biological and artificial. Despite the alien’s complete ignorance, it will be immediately struck by the appearance of a common structure that emerges in many of these flying creatures: their wings. What is particularly convincing about the wings’ role in aviation is that they appear in creatures that are built and evolved in completely different manners—say airplanes and flies. Precisely this structural convergence, despite massive divergence in make-up, is what justifies the conclusion that the wings are essential for flying. This ‘convergent evolution’ toward wings can be taken then as a strong indicator that wings play a critical role in flying even without any understanding of flight or aerodynamics. […] Such a remarkable convergence [between human and DCNN pattern-similarity structure, LK] strongly suggests that the relational geometry of the human face-selective cortex is not an accidental, developmental epi-phenomenon but rather is a structure that plays a crucial functional role in face perception.

A critic might nevertheless argue that we have not yet identified a mechanism that can effectively read the activation space and decode the firing patterns within it based on their location in the activation space. The observation of neurophenomenal similarity across numerous systems raises questions about the existence of a mechanism that can cover such a wide range of mental domains. If such a mechanism does not exist, findings of structural similarity may not indicate causally relevant neural processes.

However, this argument rests on a false premise. It is not necessary for downstream systems to read out whole neural spaces to consider neural pattern-similarity structures functional. We might understand state space structures as structural descriptions that provide a way to capture the functional similarities of a neural system’s underlying processes, while abstracting away from the specific properties of the mechanism that generates them (Kieval 2022).

To better understand the extent to which neural pattern-similarity structures exhibit functionally significant pattern similarities, it is important to investigate the relationship between state-space descriptions and the corresponding neural mechanisms that give rise to them. Structural descriptions of neural state space can be seen as abstractions from underlying processing features that capture similarities in processing without revealing the workings of the underlying mechanisms.

Let us take sensory cortex as an example. Individual neurons in sensory cortex respond selectively to particular stimuli through sparse coding, where tuning curves describe the statistical relationship between a neuron’s activity and a specific stimulus property (Dayan and Abbott 2001, Olshausen and Field 2004, Kriegeskorte and Wei 2021). When presented with an appropriate stimulus, the firing rates of tuned neurons increase, indicating that some neurons respond more strongly to specific stimuli than others. Tuning properties play a critical role in determining the activation structure of the system. An overlap of tuning curves, which results from a correlation in the firing of individual neurons, leads to an overlap in activation patterns and contributes to the distances in the state space. A high overlap of tuning curves leads to closeness in neural space.

However, the same neural pattern-similarity structure can arise from processing schemes other than tuning properties of neurons. Different neural systems can have similar neural pattern-similarity structures, despite having different underlying mechanisms (Kriegeskorte and Diedrichsen 2019). The similarity of patterns in a neural system’s state space can be influenced by various factors in addition to overlap in tuning curves, such as the architecture of neural circuits or the spatial proximity of neurons (Kriegeskorte and Wei 2021). While we can theoretically derive the activation space structure from the underlying systems’ workings, the reverse is not true: a specific neural pattern-similarity structure does not reveal the processing mechanisms that give rise to it. Thus, a given neural pattern-similarity structure can be the result of multiple possible underlying mechanisms.

State space descriptions capture functional similarities of underlying mechanisms. This is because they offer valuable insights into the relationships between the whole states of a system, even when the underlying mechanism is not fully understood. When the same parts of a system are active during the processing of different stimuli, high correlations between system states are observed. By comparing the patterns of these whole system states, we can gain insight into the overlap of processing that is activated by similar stimuli. Hence, descriptions of neural pattern-similarity structures capture the relationships between system states that arise during stimulus processing, and therefore, describing the distances between these states means describing the functional similarities of the activation patterns exhibited by underlying mechanisms when processing stimuli.

The downstream effects of these pattern-similarity structures might be understood by considering that a similar processing by upstream systems can lead to comparable effects in subsequent stages. As a result, state space descriptions can be interpreted as structural descriptions of the processing similarities of neural mechanisms performing their function, which can ultimately lead to corresponding patterns in task-related behavior.

The usefulness of abstracting from anatomical and mechanistic information varies depending on the research question. In the field of consciousness science, the abstract nature of state space descriptions is considered an advantage because the mechanisms underlying phenomenal consciousness are still poorly understood. Therefore, it is crucial to use methods that are theoretically neutral yet capable of differentiating between relevant and irrelevant neural systems. The neurophenomenal structural similarity approach allows researchers to identify neural systems associated with phenomenal structures without assuming specific underlying mechanisms. By focusing on the structure of experience and its neural coding, researchers can study the neural processes underlying quality spaces without specific assumptions, making the structural approach a powerful tool. Additional experimental evidence and modeling approaches can subsequently be applied to gain a deeper understanding of the mechanisms responsible for specific neural pattern-similarity structures.

Overall, I believe that a structuralist methodology can be effectively used despite the challenges it faces. To address criticisms of the structural similarity hypothesis, we can map specifically defined psychophysical and neural activation structures, which avoids accusations of triviality of the mapping. Importantly, this mapping can be formulated regardless of defining the representational properties of psychological or neural spaces. While there are open questions regarding the measurement of neural and qualitative structures, progress can be made by comparing different methods and formulating appropriate metrics. It is important to note that we are not seeking a precise mapping, but rather investigating the degree of structural similarity. Therefore, our methodology is holistically oriented but makes no claims about the nature or semantics of the relata. Nevertheless, a high degree of structural similarity can tell us about the relationship between individual qualitative and neural states by picking them out as coordinates in their corresponding structures. Moreover, neural pattern-similarity structures can be considered functional similarities in the processing of underlying mechanisms, even if we do not fully understand the precise mechanisms. This is particularly advantageous for the study of consciousness, where we aim to identify neuronal systems with as few assumptions about consciousness as possible.

However, the precise relationship between the structural approach and consciousness science still remains unclear. One of the methodological foundations of consciousness science is the distinction between perceptions that enter conscious awareness and those that are processed unconsciously. It is possible that neural encodings of qualitative structures play different roles in relation to this conscious/unconscious contrast. One interpretation is that they display qualitative structures that emerge only when qualities are consciously experienced. Alternatively, the structural encoding of qualitative structures may reveal neural structures of processing that are not necessarily linked to consciousness. In this case, pattern-similarity structures could arise from neural states independent of conscious processing. The mapping of qualitative structures to neural pattern-similarity structures may be independent of traditional questions of consciousness, or it may be associated with conscious awareness specifically if the neural pattern-similarity structure is influenced by conscious awareness, leading to distinctive structural features of neural processes related to consciousness.

## The specificity of neural pattern-similarity structures related to consciousness

Structuralists often conceptualize consciousness in terms of ‘specific’ neural pattern-similarity structures. Opie and O’Brien (2015: 459) posit that “certain structural properties” of neural activity give rise to conscious experience, while Malach (2021: 1) suggests that a “specific configural pattern” of activity is necessary for consciousness to emerge. However, the precise meaning of the specificity of neural pattern-similarity structures in the context of consciousness remains unclear. Do these patterns of neural activation occur solely during conscious processing? If so, can the reliable occurrence of such patterns be taken as an indicator of conscious experience? However, there is not much evidence that investigates the relationship between specific manifold structures in neural space and the differentiation between conscious and unconscious processing. Hence, we must rely on theoretical frameworks and intuitive reasoning to explore this relationship. Despite these limitations, it is important to consider the link between neural pattern-similarity structures and consciousness, as it has the potential to shed light on the neural mechanisms underlying conscious awareness. My focus is to broadly differentiate between local and non-local approaches in the field of neuroscience of consciousness, in order to provide a framework for potential interpretations. However, it should be noted that I am not asserting that localism or non-localism is necessarily tied to a specific stance on structuralism or the conscious/unconscious distinction (see later).

From the perspective of proponents of a local theory of consciousness, such as Lamme (2010) and Malach (2021), the occurrence of specific activation patterns in early sensory systems may be sufficient to account for conscious experience. In this framework, the specific structure of these patterns is closely linked to conscious processing. This is because it is plausible that the transition from unconscious to conscious processing affects neural pattern-similarity structures, as this transition is determined by characteristics of the local processing architecture. For instance, the switch from feed-forward to recurrent processing (Lamme 2010), which results in local ignitions (Malach 2021), is likely to influence neural firing patterns. Hence, when recurrent processing occurs and sensory states become consciously experienced, specific neural pattern-similarity structures emerge, referred to as “bar codes” by Malach (2021: 1), accompanying phenomenal consciousness. In other words, the structure of experience is related to specific ‘conscious modes’ of sensory processing, while ‘unconscious modes’ yield different neural pattern-similarity structures. This would potentially allow us to use the structural similarity constraint to distinguish consciousness-related processes from unconscious processing.

On the other hand, proponents of a non-local approach may contend that relying solely on neurophenomenal structural similarities to differentiate between local NCC candidates can be challenging. Although many neural systems may display an activation space resembling a particular quality space, not all corresponding neural systems are necessarily involved in the structure of conscious experience. Non-localists posit the existence of an additional mechanism beyond sensory processing that accounts for the conscious experience of otherwise unconscious sensory states. Possible additional mechanisms include global broadcasting (Mashour et al. 2020) or higher-order representation (Lau and Rosenthal 2011, Brown et al. 2019). From this perspective, the same neural pattern-similarity structures that determine conscious behavior may also account for unconscious behavior without this additional mechanism. On such an interpretation, neurally encoded structures of quality spaces are related to both conscious and unconscious perception and are not a distinct feature of phenomenal consciousness unless there is an additional mechanism in place that enables conscious experience. While the structural constraint is valuable for identifying candidate NCCs, it cannot distinguish consciousness-related from unconscious state space structures according to this account.

The question of whether the same pattern-similarity structures underlie both conscious and unconscious processing remains a topic of intense debate that is difficult to resolve empirically due to the challenges in obtaining neural signals for unconsciously processed stimuli. One potential approach to investigate the structures of unconscious processing is through the use of face stimuli, as suggested by Axelrod et al. (2015), but further research is required. While clinical cases like blindsight have been used to shed light on this question, the results are inconclusive. Some theorists argue that blindsight supports the idea that the same neural pattern-similarity structures underlie both conscious and unconscious processing. However, when considering behavioral patterns in addition to aforementioned chance performance in single tasks, it is important to note that the behavioral structure of blindsight patients across various psychophysical tasks differs significantly from that of neurotypical populations. This makes it unlikely that the same neural pattern-similarity structures underlie both conscious and unconscious processing when considering blindsight. In fact, a reviewer of this article suggested that the idea that the same psychophysical structures account for conscious and unconscious behavior is far-fetched and not worth considering from a psychophysicist’s point of view. Therefore, I take it to be more plausible that specific neural pattern-similarity structures emerge in conscious processing and determine the structure of phenomenal experience, as argued by Malach (2021) and Opie and O'Brien (2015).

However, note that while mapping qualitative structures to neural pattern-similarity structures enables the investigation of consciousness-specific properties of neural activity, the occurrence of specific neural pattern-similarity structures in consciousness does not necessarily support localist accounts of consciousness. Non-localist approaches can account for these differences by proposing that higher-order states or global broadcasting influences local processing structures. Studies on attention (Brouwer and Heeger 2013, Çukur et al. 2013) have demonstrated that postperceptual processes can influence sensory activation structures. Thus, while neural pattern-similarity structures may be necessary for conscious experience, they may not be sufficient to fully explain the nature of consciousness. Non-localist theories suggest that conscious experience may also involve higher-order states or global broadcasting, which can influence the processing of local neural activity.

I hope to have convinced the reader that considering neural pattern-similarity structures that correspond to conscious processing is important for further developing neuroscientific theories of consciousness. If conscious experience is tied to modulations in neural pattern-similarity structures, phenomenal consciousness might be statistically conceptualized as a moderator that influences differences in neural pattern-similarity structures of sensory systems. Therefore, investigating these modulations through consciousness-related processes may offer new insights into the neural basis of consciousness and provide opportunities to test and evaluate various theories. Methodologically, the structural approach allows us to study the relationship between conscious experience and underlying neural activity and could help to refine our understanding of the NCC.

## Conclusion

The structural approach presents a promising avenue for investigating the neural basis of consciousness and can withstand various forms of criticism. Methodological criticisms, such as interindividual comparisons of neural pattern-similarity spaces and measurements of phenomenal structures, can be addressed through careful experimental design and progress in methodology. Other criticisms, such as those related to holistic definitions and questions about representation, do not directly challenge the mapping from phenomenal structures to neural activation structures. Although the question of the functionality of neural state space structures is complex, it can be resolved by understanding these structures as displaying functional similarities of underlying processes that abstract from the anatomical and mechanistic properties of the system.

By identifying neural systems that exhibit activation patterns that mirror the structure of phenomenal similarities, the structural approach sheds light on the neural processes that process stimuli in a manner that matches the structure of appearances of these stimuli. As a complement to the contrastive approach, the structural approach provides an alternative methodology for uncovering the NCC. By establishing a close relationship between the structure of experience and neural activation patterns, the structural approach holds great potential for further development in consciousness science.

## Data Availability

No data are available as this is a purely conceptual paper
